# Blocking epithelial-to-mesenchymal transition in glioblastoma with a sextet of repurposed drugs: the EIS regimen

**DOI:** 10.18632/oncotarget.18337

**Published:** 2017-06-01

**Authors:** Richard E Kast, Nicolas Skuli, Georg Karpel-Massler, Guido Frosina, Timothy Ryken, Marc-Eric Halatsch

**Affiliations:** ^1^ IIAIGC Study Center, Burlington, VT, USA; ^2^ INSERM, Centre de Recherches en Cancérologie de Toulouse, CRCT, Inserm/Université Toulouse III, Paul Sabatier, Hubert Curien, Toulouse, France; ^3^ Department of Neurosurgery, Ulm University Hospital, Albert-Einstein-Allee, Ulm, Germany; ^4^ Mutagenesis & Cancer Prevention Unit, IRCCS Azienda Ospedaliera Universitaria San Martino, IST Istituto Nazionale per la Ricerca sul Cancro, Largo Rosanna Benzi, Genoa, Italy; ^5^ Department of Neurosurgery, University of Kansas, Lawrence, KS, USA

**Keywords:** AMPK, EIS regimen, EMT, glioma, glioblastoma

## Abstract

This paper outlines a treatment protocol to run alongside of standard current treatment of glioblastoma- resection, temozolomide and radiation. The epithelial to mesenchymal transition (EMT) inhibiting sextet, EIS Regimen, uses the ancillary attributes of six older medicines to impede EMT during glioblastoma. EMT is an actively motile, therapy-resisting, low proliferation, transient state that is an integral feature of cancers’ lethality generally and of glioblastoma specifically. It is believed to be during the EMT state that glioblastoma’s centrifugal migration occurs. EMT is also a feature of untreated glioblastoma but is enhanced by chemotherapy, by radiation and by surgical trauma. EIS Regimen uses the antifungal drug itraconazole to block Hedgehog signaling, the antidiabetes drug metformin to block AMP kinase (AMPK), the analgesic drug naproxen to block Rac1, the anti-fibrosis drug pirfenidone to block transforming growth factor-beta (TGF-beta), the psychiatric drug quetiapine to block receptor activator NFkB ligand (RANKL) and the antibiotic rifampin to block Wnt- all by their previously established ancillary attributes. All these systems have been identified as triggers of EMT and worthy targets to inhibit. The EIS Regimen drugs have a good safety profile when used individually. They are not expected to have any new side effects when combined. Further studies of the EIS Regimen are needed.

## INTRODUCTION

Epithelial to mesenchymal transition (EMT) refers to a transient process where flat, sessile, mutually adherent epithelioid cells take on a rounded, non-adherent, motile mesenchymal shape and behavior [1-4]. EMT is engaged by normal cells during wound healing and is identified in cancer generally. The reverse, less transient state and process, mesenchymal to epithelial transition (MET), also occurs. Both processes are features of robust or aggressive cancer growth [5]. Cells post-EMT tend to be invasive but proliferation-restricted. Cells post MET tend to be proliferative but have limited invasiveness [1-4]. Table 1 lists some of the surface markers and cell characteristics commonly used to define, and are associated with, the two phenotypic states. EMT has been demonstrated in and is central to glioblastoma (GB) pathology [6]. In a landmark immunohistochemical study looking at paired primary and recurrent GB, Kubelt et al showed high expression of vimentin, TGF-beta, beta-catenin and fibronectin- all EMT markers- in both primary tumor and in recurrence tissues [6].

**Table 1:** Some of the biochemical marker and phenotypic changes characteristic of and concomitant to transformation of a cancer cell from epithelial to a mesenchymal phenotype.

marker / mediator	epithelial state	mesenchymal state
E-cadherin	increased	decreased
N-cadherin	decreased	increased
ZO-1	increased	decreased
occludin	increased	decreased
vimentin	decreased	increased
fibronectin	decreased	increased
MMP-2	decreased	increased
MMP-9	decreased	increased
**phenotype**	**epithelial state**	**mesenchymal state**
motility	sessile	motile
shape	elongated	rounded
adherence	adherent to neighbors	non-adherent to neighbors
invasion	non-invasive	invasive
proliferation	high	low
microtentacles	absent	present *

In this paper we propose a combination of 6 repurposed, already-marketed drugs in order to inhibit EMT during primary treatment of GB. As we will show, this EMT inhibiting sextet, the EIS Regimen, is predicted to be safe and carry low risk of side effects. The EIS Regimen uses ancillary attributes of older, already-marketed drugs to block individual elements of EMT triggered by our current standard treatment of GB. EIS uses the antifungal drug itraconazole to block Hedgehog signaling (Hh), the antidiabetes drug metformin to block AMP kinase (AMPK), the analgesic drug naproxen to block Rac1, the anti-fibrosis drug pirfenidone to block transforming growth factor-beta (TGF-beta), the psychiatric drug quetiapine to block receptor activator NFkB ligand, (RANKL) and the antibiotic rifampin (also called rifampicin) to block beta-catenin nuclear functions- all by their previously established ancillary attributes. Hh, AMPK, Rac1, TGF-beta, RANKL, and beta-catenin have all been identified as triggers to EMT. There doesn’t seem to be a single path to EMT development. The multiple triggers initiating EMT that are discussed in this paper might have a common denominator but such hasn’t been recognized yet and may not exist.

Glioma initiating cells (GIC, also called glioma stem cells) is an evolving concept that refers to minor subpopulations within a tumor that i] are relatively quiescent, ii] are relatively resistant to cytotoxic chemo- and radiotherapies compared to the bulk population, iii] are the residual post-treatment populations from which recurrent tumor regrows, iv] that need fewer cells to establish xenograft growth compared to the bulk population, and v] display in some cases ability to undergo asymmetric cell division where one daughter cell retains stem cell characteristics, the other daughter cell has characteristics of the non-stem majority population.

Concepts and definitions of EMT, as for concepts of GIC, are both in evolution and defined in general outline only [7-11]. Intermediate and mixed forms are recognized but without assuming strictly dichotomous categories, the GIC subpopulation tends to reside in cells with more mesenchymal post-EMT attributes [7, 12, 13]. Some evidences also show that GIC present high expression of EMT markers involved in migration and invasion, such as the matrix metalloproteinases (MMP), particularly MMP-2 and MMP-9 [14, 15]. This enrichment in proinvasive/migratory genes confers GIC stronger invasive and infiltrative capacities. Both EMT and GIC may be reversible processes. Mesenchymal transformed cells revert to an epithelial form and marker status, (undergo MET) whilst non-GIC cells can assume cancer initiating properties and gain relevant markers. (Note that abbreviation MET also can refer to the unrelated cMET, the cell surface receptor for hepatic growth factor). These different GIC coexist within the same tumor and can switch from one invasive state (mesenchymal) to a proliferative one (epithelial) to allow tumor expansion and growth [16].

The growth enhancing role of EMT in GB has been thoroughly reviewed in 2016 by Iser et al [17]. The development of detyrosinated alpha-tubulin microtentacles occurring during EMT facilitates tumor cell insinuation between endothelial cells, starting tumor cell journey to distant metastasis sites [18] or in the case of GB, migratory spreading within the brain [19]. Through a variety of mechanisms, among which reduced proliferation may play a major role, EMT makes cancer cells more resistant to our traditional, currently available, cytotoxic chemotherapy [20]. Entering EMT is a major resistance mechanism for GB resistance to erlotinib [21] for example. Importantly, circulating tumor cells from which metastases are derived, are mostly cells that have undergone EMT [22-24].

Table 1. lists the protein expressions and behavioral attributes associated with the epithelial or mesenchymal state. Although the Table lists attributes of the polar states, presence of intermediate states is the rule.

E-cadherin is a multifunctional, highly phosphorylated outer cell membrane protein active in securing epithelial-to-epithelial cell adherence. E-cadherin undergoes a shift in molecular weight going from 125 to 115 kDa during iron overload, reversing back to 125 kDa after Fe++ chelation [25]. The intracellular domain of E-cadherin binds beta-catenin. Thus, less surface E-cadherin, often mirrored by increase of N-cadherin [26, 27], results in less adherence to neighbor cells facilitating tumor cell spreading. It is also coupled to loss of intracellular beta-catenin sequestration, making increased beta-catenin available for transport into nucleus where it is a malignancy-associated transcription factor. An interesting question is whether E-cadherin changes reflect or contribute to mediating (or both) EMT [28]. Vimentin is one of several markers in tissues undergoing EMT, as listed in Table 1. Immunohistochemical and mRNA study of GB biopsy tissues showed that high vimentin expression was associated with significantly shorter survival compared with GB with lower vimentin expression [29].

Among the multiple regulators of, and triggers to, EMT in GB is also epigenetic regulation by small non-coding RNAs or microRNAs (miRNA, a sequence of 20 to 25 nucleotides). miRNA have already been described to play a major role in GB growth, proliferation, migration and invasion processes [30]. More recently and more specifically, microRNAs have also been shown to play various roles in GB’s EMT process generally, and specifically by increasing transforming growth factor-beta (TGF-beta) [31, 32], a fact of particular importance in our use of TGF-beta inhibitor pirfenidone (vide infra, Section 3.a. on pirfenidone).

It would be wrong to view EMT as a single core element of malignancy. Both EMT and MET are crucial attributes for vigorous malignant growth. Without either process cancer would be easier to cure with modern techniques, both processes must be addressed for effective treatment. EMT is crucial for release of malignant cells to blood or lymph, but MET is crucial for metastasis establishment and growth [3], corresponding to the oncology aphorism “go or grow”. Otherwise said, while EMT enhances cells leaving the primary tumor mass, MET is the process that enhances distant colony establishment and growth [3, 33]. GB patients have readily identifiable circulating GIC. These have markers and characteristics of post-EMT cells [34] indicating EMT as an active process in GB. Cancer cell epithelial or mesenchymal state seems not to be strictly binary states. Intermediate states are the rule rather than the exception. To summarize, EMT-MET phenotypic shuttling is a central and defining feature of GB, with GIC residing in two phenotypic gradients each of which culminates in one specific pole : higher proliferative activity with angiogenesis (epithelial state) or higher migratory activity with attenuated mitosis (mesenchymal state) [35].

Multiple triggers to EMT initiation are present as part of the natural biology of GB: hypoxia, inflammation, acidic milieu, epidermal growth factor (EGF) signaling are examples [35]. Current treatments with cytotoxic chemotherapy (temozolomide in the case of GB), surgical tissue disruption and radiation have all been recognized as triggers for remaining viable cells to enter EMT, details given in Section 2. below.

GB heterogeneity of driver mutations [36, 37] extends likewise to heterogeneity of EMT drivers. In examining pancreatic adenocarcinoma intratumoral heterogeneity Dembinski and Krauss found large but incomplete overlap between slowly cycling cell subpopulation and those expressing commonly accepted stem cell markers (e.g. ALDH, CD44, CD133) and behaviors [38]. Most enlightening was their finding that EMT characteristics were stimulated largely by Hh and TGF-beta signaling, and crucially such stimulation and marker changes were accompanied by decreased epidermal growth factor receptor (EGFR) expression. This implies a dynamic shuttling or see-saw process where TGF-beta and Hh signaling increase EMT but decrease EGFR dependency, partially explaining erlotinib failures.

The hypoxic microenvironmental islands characteristic of GB are drivers of EMT. Tumor hypoxia, via the Hypoxia Inducible Factors (HIF-1α and HIF-2α), directly or indirectly control the expression of several EMT transcriptional regulators such as Snail, Slug, Twist1 or ZEB1/2 [39]. Aberrant reduced expression of E-cadherin, the HIF or the EMT regulators is correlated with more aggressive tumors and poor prognosis [12, 13]. Hypoxia also results in the recruitment of myeloid cells (e.g. macrophages and neutrophils), which can secrete TGF-beta, and other signaling proteins resulting in subsequent EMT promotion [40]. Of note, these secreted factors are known HIF inducers in GB [41] thus adding another level of complexity to EMT initiation in GB.

Additionally, hypoxia-induced EMT particularly occurs in areas of CXCL12 stimulation of outer cell membrane CXCR4 [42] with increasing N-cadherin and matrix metalloproteinase-9 (MMP9) [43]. This may be relevant to the GB remission case reported by Rios et al [44]. These authors reported an unusual durable response in a GB patient treated with adjuvant therapy consisting of temozolomide and a weekly dose of plerixafor, a CXCR4 inhibitor, together with lapatinib, high dose metformin and niacinamide [44]. What role the individual components played is unknown, but high dose metformin use may be noteworthy (Section 3.f.).

Tabouret et al have shown in paired primary and recurrent resection tissues that post-radiation recurrent GB had upregulated CXCR4/CXCL12 signaling [45]. However, a clinical trial of plerixafor in GB failed to show benefit [ClinicalTrials.gov Identifier:NCT01339039]. Data by Pharm et al may hint at the reasons why this trial failed despite the durable response described by Rios et al using plerixafor and a number of clinical and preclinical reports confirming the importance of CXCR4/CXCL12 in GB growth and invasion [46]. Pharm et al found that CXCR4/CXCL12 signaling in GB exists in a mutually inverse relationship (see-saw, reciprocal) with signaling of vascular endothelial growth factor receptor (VEGFR) [46].

Since several reports show metformin-mediated reduction of VEGFR function in both preclinical [47-49] and in mice fed a high fat diet and clinically in polycystic ovarian syndrome (PCOS) and type 2 diabetes settings [50-52], inhibiting both systems- VEGFR function by metformin and CXCR4/CXCL12 by plerixafor, might be required to achieve a durable antitumor response.

To summarize, preventing EMT during GB treatment might be a constructive step. Below are a sextet of already-marketed drugs with evidence that they inhibit in coordinated fashion one or another aspect of the EMT process. They are designed to be given all together to GB patients, during and alongside the standard therapeutic temozolomide based protocol for glioblastoma [53]. In Section 2. below we present data indicating that all 3 glioblastoma treatment modalities- primary resection, radiation as well as temozolomide provoke EMT entry in glioblastoma, EIS Regimen might well be started even before biopsy and continued through end of temozolomide.

## TREATMENT TRIGGERS EMT

It should not be considered odd that our mainstay current treatment modalities for cancer- cytotoxic chemotherapy, radiation, and resection- have all been shown to enhance or trigger EMT and thus have tumor growth-promoting aspects. In chess, fencing, or other forms of combat, including other fields of medicine, actions that in sum further our goals and prolong life, also contain within those actions elements that work against our goal. The chess aphorism “All moves create weaknesses and strengths” applies to all areas of medical intervention. So our job is to assess that balance in deciding to offer a treatment. The EIS Regimen is designed to run alongside all phases of initial GB standard treatments - surgical resection, temozolomide, and radiation - with the purpose to diminish the EMT triggered by treatment.

### Surgery induces EMT

That surgery induces EMT in tissues within the operative field should not be surprising in that EMT is an integral feature of normal wound healing and is triggered by any tissue integrity disruption [54].

We give here a few examples of tissue disruption by surgery or even simple needle core biopsy triggering EMT. Breast cancer fine needle biopsy engages EMT in mice [55]. Of potential clinical concern, incision biopsy of oral squamous cell carcinoma causes tumor-associated macrophages to produce increased local TGF-beta [56, 57] that in turn contributes to local immunosuppression and EMT as outlined in Section 3.a. below. Similarly, during human breast cancer diagnosis, the site of needle biopsies show recruitment of inflammatory cells that cause an increased proliferation rate of surrounding breast cancer cells [58]. Standard transrectal ultrasound guided prostate biopsy results in detectable prostate cancer cells in the circulation in half of patients [59]. Incisional biopsy of oral squamous cell carcinoma results in 16% of patients having post-biopsy circulating tumor cells indicative of post-EMT status [60] whereas excision wide enough to not disrupt the tumor tissue integrity did not result in post-operative circulating tumor cells [61 ].

Conclusion: GB tissue disruption might engage EMT in scattered residual cells.

### Cytotoxic chemotherapy induces EMT

Temozolomide enhances migration and EMT in some- but not all- ex vivo glioma cell lines and fresh patient glioblastoma cells [62]. Temozolomide exposure induces several EMT markers including high vimentin, TGF-beta, beta-catenin, and fibronectin in T98G glioma cells [6]. In testing breast cancer cells for doxorubicin cytotoxicity, 2 independent groups found that those cells not killed by doxorubicin were induced to undergo EMT and those post-EMT cells were relatively doxorubicin resistant [63, 64]. Similar doxorubicin induction of EMT was confirmed in triple negative breast cancer [64] and in hepatocellular carcinoma cells [65, 66], colon cancer cells [67] and non-small cell lung cancer cells [68]. Gastric cancer cells exposed to sublethal doxorubicin were phenotypically mesenchymal and overexpressed vimentin, twist and beta-catenin [69]. 5-FU induced EMT and increased TGF-beta in colon cancer cells [70]. Most relevant to our subject, doxorubicin also induces EMT in glioma cells [71]. KM12L4 and HT29 colon cancer cell lines exposed to oxaliplatin lose E-cadherin, increase vimentin, and become motile while becoming more resistant to oxaliplatin [72]. During chronic exposure to paclitaxel, ovarian cancer cell lines take on a mesenchymal phenotype, lose E-cadherin and increase expression of vimentin and fibronectin [73].

Conclusion: Current cytotoxic chemotherapy drugs tend to engage EMT programs.

### Radiation induces EMT

We have many examples where radiation that didn’t kill exposed cells, triggered them to undergo EMT. Radio-resistant glioma cells exhibit a specific signature enriched in genes belonging to the EMT process [74]. Reviews in 2014 and again this year (2017) of collected data from multiple different cancers make a strong argument that a) ionizing radiation induces increased metastasis potential and enhanced invasiveness of surviving cancer cells and b) it does so via induction of EMT programs [75, 76].

Empirically, radiation enhancement of migration and triggering of EMT specifically in GB can be readily demonstrated [77-83].

Radiation enhanced centrifugal migration of U87 glioma cells implanted in mice and also increased brain levels of IL-1, PGE2, IL-6, and TNF-alpha (see Section 3.d below) [77]. U251 glioma cells were triggered to enter EMT and migrate after exposure to X-rays [79]. Non-gamma ionizing radiation at intermediate doses also triggered glioma cells to migrate and express EMT markers. At doses above intermediate, cell death occurred, below intermediate dose failed to induce EMT [80]. That “cells surviving radiation can become more aggressive and invasive” was confirmed in 2 independent studies GB [81, 82]. Most instructive was the study of Desmarais et al with radiation of rats with orthotopic implanted glioma cells. Radiation enhanced the implanted glioma cells’ migration and shortened survival compared to control rats implanted with the same glioma cells but not radiated [83].

Similar ionizing radiation induction of migration and other EMT attributes can be demonstrated in other cancers. For example non-lethal X-radiation at 0.4 Gy/min induced migration, individual cell morphology changes typical of EMT, strong upregulation of EMT mediating transcription factors Snail and Twist, decreased E-cadherin and increased vimentin and fibronectin in MCF-7 breast cancer cells [84]. Nearly identical increases in motility and phenotype changes with concomitant decreases in E-cadherin and increases in vimentin and fibronectin have been described in irradiated colorectal cancer cell lines [85]. Human non-small cell lung cancer cells surviving 10 Gy radiation showed increased motility and increased MMP-2/-9 [86]. Dramatic demonstration of the development of microtentacles after colon cancer cell exposure to 5Gy X-ray can be seen in scanning electron micrographs [87]. Endometrial cancer cells [88] and squamous esophageal cancer cells [89] take on typical EMT phenotypic changes and increase migration after gamma radiation. Biopsy from the radiated field in rectal cancer shows regions of cells having undergone EMT with elevated Slug, Snail, and vimentin [90].

Two breast, 2 colon and 2 lung adenocarcinoma cell lines subjected to 2 Gy increased vimentin and motility while taking on typical mesenchymal morphology [91]. Altogether, these data strongly suggest that GB cell response to radiation contribute to the EMT process and the acquisition of an invasive and aggressive phenotype.

Conclusion: So while radiation of the post-resection area improves overall survival, it also induces EMT in the few surviving cells that have migrated deep into brain, setting the stage for later aggressive regrowth of tumor. Or as Niccolo Machiavelli (1469 - 1527) said in 1513 “People should either be caressed or crushed. If you do them minor damage they will get their revenge; but if you cripple them there is nothing they can do. If you need to injure someone, do it in such a way that you do not have to fear their vengeance.” EIS Regimen was crafted with that in mind.

## THE EIS REGIMEN: EMT TRIGGERS, MAINTENANCE FACTORS, AND 6 CURRENTLY AVAILABLE DRUGS TO INHIBIT BOTH

The EMT-triggering targets presented here have been selected based in part on a) their having readily available repurposed drugs to inhibit them that b) are already FDA, EMA, ANSM (French FDA equivalent) and Health Canada approved for use in humans. The guiding precept was that we must treat today’s disease in today’s patient with the medicines and tools we have available today. Additionally, the growing cost of medical care worldwide, particularly in oncology is a further incentive to repurpose already marketed, clinically used non-oncology drugs to augment effectiveness of traditional oncology drugs. The EIS Regimen uses medicines that have been well-proven to be safe and effective in their original indication. If they will be likewise effective as adjunct in treating GB must be tested.

### Inhibiting TGF-beta: pirfenidone

Of all the EIS Regimen drugs, the one with the strongest evidence for benefit during GB treatment is pirfenidone. Pirfenidone is a 185 Da drug first approved to treat idiopathic pulmonary fibrosis [92, 93]. It has since been investigated in treating hepatic fibrosis and other diseases where excessive fibrosis is a problem. It is well-tolerated, side effects do not usually necessitate stopping. Side effects of pirfenidone tend to be gastrointestinal and generally mild - nausea, dyspepsia, vomiting, anorexia, or less commonly rash - but all without long term sequelae [94, 95].

Pirfenidone works in part by blocking TGF-beta signaling [92, 94, 96]. TGF-beta is a 25 kDa signaling protein proteolytically clipped from a large precursor protein. TGF-beta signaling is a major driver of EMT in cancer generally [97, 98] and in GB’s EMT specifically [12, 13, 99-102].

TGF-beta is a facilitating element of many cancers by promoting angiogenesis, immune suppression and EMT induction [103]. After cell surface binding to its heteromeric receptor complex serine/threonine kinase, multiple processes including activation of nuclear transcription factors and cytoplasmic cytoskeleton changes - are set in motion and initiate and sustain EMT [104]. Multiple intermediary transcription factors activated by TGF-beta including Snail, Slug, Twist, SIP1/ZEB, and E47, down regulate E-cadherin expression and engage EMT related programs [105]. Chronic TGF-beta-mediated stimulation in both cancer or pathologic wound healing may drive excess extracellular matrix and collagen synthesis and deposition [106]. TGF-beta induces phosphorylation of Smad, p38, Akt, and smooth muscle actin and collagen mRNA levels - all elements of EMT- in normal pulmonary fibroblasts during development of pulmonary fibrosis. All these changes can be inhibited by pirfenidone [107]. Pirfenidone inhibits experimentally TGF-beta driven migration and EMT in normal epithelia [108, 109].

Experimental unilateral ureteral obstruction in rats results in tubulointerstitial changes characteristic of EMT- increased TGF-beta1, type III collagen, α-SMA, S100A4, fibronectin and reduced expression of E-cadherin. Pirfenidone treatment tended to diminish these changes [110], reducing obstruction provoked EMT and renal fibrosis. Pirfenidone also inhibited TGFbeta driven collagen and fibronectin overproduction in non-small cell lung cancer cell lines [111]. In dextran-induced murine colitis, pirfenidone reduced the elevated TGF-beta mRNA, SMAD signaling, and consequent fibrosis [112]. Pirfenidone inhibited migration of ex vivo TGF-beta stimulated nasal polyp fibroblasts concomitantly with blocking TGF-beta-induced SMAD phosphorylation [104].

These data are effects we expect pirfenidone to likewise do during GB treatment. Of added benefit during GB, pirfenidone has activity also in inhibiting TNF-alpha and platelet derived growth factor (PDGF) [96], both growth factors implicated in driving GB growth. TGF-beta triggers prostate cancer cell motility and enhances bone marrow cell recruitment to the growing tumor stroma. [113, 114].

An early (1994) study showed that while TGF-beta enhanced invasiveness and migration of glioma cells, it also decreased mitotic rate, indicative of EMT induction [115]. A recent study (2016) showed that an experimental peptide TGF-beta inhibitor, P144, decreased GB invasion, migration, and increased apoptosis [116]. microRNA-564 mediated decrease in TGF-beta inhibited proliferation and invasiveness of U87 glioma cells [Jiang]. Phytoderived delphinidin inhibited TGF-beta-mediated increases in fibronectin and migration of U87 glioma cells by interfering with translation factor SMAD and others, downstream from TGF-beta receptors [117]. None of the latter 3 anti-TGF-beta agents- P114 peptide, microRNA-564, or delphinidin- are marketed pharmaceuticals available to treat GB patients today. Pirfenidone is.

Ohshio et al have shown that blocking TGF-beta release from lung cancer cells reduced their motility and invasiveness, increased their expression of E-cadherin and reverted their morphology to epithelial from mesenchymal but had no effect on the cells viability [118]. Similarly, in hepatocellular carcinoma TGF-beta synthesized and released by tumor infiltrating monocytes, drives the proper carcinoma cells’ EMT [119]. A parallel relationship between monocyte lineage cells and malignant phenotype has been described in GB as well [120].

Circulating IL-6 and TGF-beta levels are elevated in chronic hepatitis C. After 2 years treatment with pirfenidone (400 mg, 3 times daily), levels of both cytokines were lowered by ∼ 50% [121]. See Section 3.d. below for discussion on advantages of diminishing IL-6. Retinal pigment epithelial cells after exposure to TGF-beta undergo phenotypic, biochemical marker and enhanced migration changes typical of EMT. Exposure to pirfenidone prevented these effects of TGF-beta by preventing SMAD translocation to nucleus [122]. Pirfenidone inhibits TGF-beta increases after bleomycin exposure in rats as well [123]. TGF-beta signaling in connection with another EIS drug, metformin, is discussed under section 3.f.

Pirfenidone was first suggested as a potentially useful treatment adjunct for GB in 2007 [124]. In this Section we detailed further evidence that TGF-beta is an important GB growth factor and major trigger for GB cells’ EMT. Inhibition of TGF-beta expression in malignant glioma cells by pirfenidone is worth investigating as adjunctive treatment alongside of current primary GB treatment with resection, temozolomide and radiation.

### Inhibiting RANKL: quetiapine

Quetiapine is a 383 Da generic psychiatric drug originally marketed to treat psychoses. It has since been found to have other attributes, including norepinephrine reuptake inhibition and strong antihistamine effects leading to adjunctive use in treating depression and insomnia, respectively.

Our addition of quetiapine to the EIS Regimen hinges on a single report showing that quetiapine inhibits the Receptor Activator of Nuclear Factor Kappa B Ligand (RANKL) signaling [125]. Secondary consideration recommending quetiapine’s addition was that it is well tolerated in a non-psychiatric population- it will not add to side effect burden.

RANKL becomes overexpressed in parallel with vimentin and N-cadherin during TGF-beta-induced EMT in prostate cancer cell lines [126]. RANKL signaling may be more of a mediator of EMT behaviors than a trigger to initiate or maintain EMT programs. An interesting dataset connects RANKL to EMT. RANKL-activation of RANK increased expression of vimentin, N-cadherin, Snail, and Twist, decreased the expression of E-cadherin and drove EMT in normal breast acinar cells and a number of cancers including breast cancer [127], hepatocellular carcinoma [128, 129], endometrial [130, 131], lung [132] and prostate cancer [126].

GB secreted RANKL enhances GB invasive motility in part by paracrine signaling to surrounding non-malignant astrocytes, triggering these astrocytes to secrete TGF-beta that in turn facilitates centrifugal glioma cell migration/invasion [133]. Thus the EIS Regimen attempts to undermine the RANKL and TGF-beta mediated growth enhancing cycle between glioma cell and surrounding normal astrocytes by coordinated inhibition with quetiapine and pirfenidone.

### Inhibiting beta-catenin-mediated canonical Wnt signaling: rifampin

Rifampin (synonymous with rifampicin) is an 823 Da antibiotic introduced to clinical use in the 1960’s, used today largely for treating tuberculosis, Hansen’s disease, treatment resistant Staphylococcal, and H.pylori infections [134]. Rifampin functions in EIS Regimen to a) reduce microglial activation and b) inhibit Wnt/beta-catenin signaling (these 2 attributes might be related).

a) Rifampin is neuroprotective by inhibiting microglial activation and is in active study in Parkinson’s disease on that basis [135, 136].

b) Wnt signaling forms an important growth element in many cancers [137, 138] including in GB [139, 140]. Wnt signaling is divided into canonical and non-canonical [141]. Canonical Wnt signals through the 92 kDa intracellular transcription factor beta-catenin. After Wnt ligand binding, beta-catenin is translocated to the nucleus where it binds to cognate DNA recognition sequences and promotes transcription of a number of target genes. In the absence of Wnt ligand, beta-catenin is degraded via the ubiquitin-proteasome pathway [142]. Poly-phosphorylation and poly-ubiquitination mark beta-catenin molecules doomed for proteasomal destruction [142]. beta-catenin can also be sequestered in cytoplasm by binding to the intracellular domain of E- or N-cadherin and therefore become unavailable to serve as a transcription factor in nucleus [137-140].

Experimental beta-catenin agonists enhance glioma cell migration that is impaired in the presence of beta-catenin inhibitors [143]. For instance, tetrandrine is a 623 Da phytoderived molecule that inhibits glioma cell (and urothelial cancer cell) migration by blocking beta-catenin translocation to the nucleus [144, 145]. EIS Regimen uses rifampin to do the same.

In early 2016, an unusual case of retarded progression in a non-small cell lung cancer patient being treated for tuberculosis with rifabutin led Li et al [146] to experimentally investigate the anti-cancer effect of rifabutin. Rifabutin is an 847 Da antibiotic closely related to the older rifampin. It was found that rifabutin prevented Wnt-mediated beta-catenin’s protection from proteasomal degradation and by this mechanism inhibited non-small cell cancer’s growth both *in vitro* and in an *in vivo* xenograft model [146]. Since rifabutin doesn’t cross the BBB but the closely related rifampin does, the latter would be preferable in inhibiting canonical Wnt/beta-catenin nuclear signaling in GB. Canonical Wnt is a convergence point for many signaling systems triggering EMT, promoting glioma growth, and driving centrifugal glioma cell motility [147-156].

beta-catenin signaling upregulation forms part of glioma cells’ development of temozolomide resistance [151]. Upregulation of beta-catenin signaling as a response to ionizing radiation is a component mediating GB’s EMT response after radiation exposure [154] (as discussed in Section 2.c. above). Experimental, non-marketed beta-catenin signaling inhibitors inhibited experimental glioma’s growth [145, 149, 152, 156].

Inhibiting the canonical Wnt/beta-catenin signaling with rifampin is a worthwhile strategy to explore in GB treatment.

### Inhibiting Rac1-mediated non-canonical Wnt signaling and IL-6: naproxen

Non-canonical Wnt signaling occurs without participation of beta-catenin [140, 141]. Rac1 (acronym for ras-related C3 botulinum toxin substrate 1) is a 21 kDa GTPase that serves as a transducer, an intracellular link between non-canonical Wnt signaling events and end-effects in the nucleus [139-141]. Experimental Rac1 inhibition potentiates cytostasis and cytotoxicity by imatinib, gefitinib [157] and erlotinib in GB [158]. Experimental Rac1 inhibition inhibits colon cancer cells’ migration as well [159].

Naproxen is a 230 Da, commonly used cyclooxygenase (COX) inhibitor, marketed as an analgesic. Cerebrospinal fluid (CSF) naproxen levels not higher than 3 microM may be achieved under usual treatment conditions. Oprea et al have reported EC_50_ for Rac1 of R-naproxen being 18 microM [160]. Given the achievable brain tissue levels of naproxen as compared to Rac1 inhibition data by Oprea and coworkers, naproxen would not be the ideal Rac1 inhibitor. Albeit something more potent would be preferable and medicinal chemistry studies aimed to development of more effective Rac1 inhibitors are warranted, preclinical investigations on the potential therapeutic effect of naproxen on GIC-driven orthotopic GB may provide useful indications on the role of Rac1 in GB’s EMT. Again, although not ideal we must fight GB with the tools we have at hand.

IL-6 is a cofactor promoting GB’s EMT. Collected data on growth promotion and apoptosis inhibition functions of IL-6 in GB up to 2011 was reviewed by Yeung and coworkers [161 ] Since then circulating IL-6 was found elevated in GB [162] as was CSF IL-6 level [163].

COX inhibitors tend to reduce IL-6 by decreasing intracellular cAMP [164-166]. Specific recent examples of this: naproxen clinically reduced IL-6 in IL-6-overproducing pheochromocytoma [167], in sera of patient with osteoarthritis [168], in subchondral osteoartrthritis osteoblasts [169], and in osteoarthritis patient synovial cells ex vivo [170]. Also in rheumatoid arthritis plasma and synovial fluid show increased IL-6 that is decreased by naproxen treatment [171]], and in a cardiac myxoma patient with elevated IL-6 [172]. In accord with what we know of intracellular control of IL-6 generation, COX inhibition generally reduces circulating IL-6. IL-6 suppression may be a worthy target during GB treatment.

It is established that GB cells synthesize IL-6 and it functions as a significant growth and migration enhancing factor [173-194].

Selected examples: Exogenous IL-6 enhanced glioma cell migration *in vitro* [173]. A pharmaceutical monoclonal anti-IL-6 antibody inhibited glioma cell proliferation [174], radiation induced increased IL-6 synthesis (cf Section 2.c. above) [176] contributing to glioblastoma related reduced immune function. There is an IL-6 based positive feedback loop in glioblastoma where extracellular IL-6 results in intracellular STAT3 phosphorylation (activation) that in turn upregulates glioblastoma cells’ IL-6 synthesis [181]. Glioblastoma show IL-6 gene amplification and patients with greater degree of amplification have shorter overall survival [184, 185]. Peripheral blood mononuclear cells from GB patients secrete abnormally large amounts of IL-6 as well [190]. Immunohistochemical IL-6 staining of GB tends to be heavier in perinecrotic areas [190].

In a parallel fashion reminiscent of radiation induction of EMT (see Section 2.c. above), IL-6 synthesis by GB cells was stimulated by intermediate dose radiation whereas low or high dose radiation did not increase IL-6 [180]. Earlier studies have shown that GB cells increase IL-6 synthesis and release after sublethal radiation and that this release increases with time over the first post-radiation day [191]. Aspirin, a balanced COX1-2 inhibitor reduced GB cell synthesis of IL-6 and induced cell death, an effect that was counteracted by adding exogenous IL-6 [192].

However, 2 clinical failures to find naproxen lowering of IL-6 (after spinal surgery and in rheumatoid arthritis) [195, 196] are of concern. This matter mandates monitoring IL-6 during EIS Regimen to determine if naproxen is in fact working as intended.

The observation that IL-6 can transactivate EGFR in GB in the absence of any EGFR ligand in GB [197], may help to explain why the EGFR inhibitor erlotinib was not effective clinically in treating GB tumors even when these are growth suppressed by erlotinib *in vitro* and overexpress EGFR *in vivo* [198].

In conclusion, limiting IL-6 function seems to be an eminently worthwhile goal in treating glioblastoma. Naproxen might not be an ideal drug to use for that, but naproxen is cheap, readily available, well-tolerated, and the drug we have today.

### Inhibiting hedgehog: itraconazole

Itraconazole, an old 706 Da antifungal drug, is undergoing a renaissance of interest for its anticancer effects [199, 200]. The primary mode of anti-cancer action is inhibition of Hh signaling [199, 200]. Hh is an important driver of GB growth [201-207]. Hh signals through intracellular transcription factor Gli [205, 206]. Gli1-driven transcription induces EMT via induction of Snail, a repressor of E-cadherin in many other cancers.

Itraconazole inhibits release of Gli1 thus keeping it sequestered in the cytoplasm [205]. GB patients with low Gli1 expression had longer overall survival [202]. The experimental Hh signaling inhibitor cyclopamine, or suppressing Gli1 expression by using siRNA interference led to decreased cell proliferation and enhanced apoptosis in U87 glioma cell line [208].

In preclinical studies itraconazole inhibition of Hh signaling inhibited growth of breast cancer [209], melanoma [210], and endometrial cancer [211].

### Activating AMPK: metformin

Metformin is a 129 Da drug in common first line use when type 2 diabetes is diagnosed [212 ]. In 2013, metformin was called “a rising star to fight (EMT) in oncology” by Barriere and coworkers who recounted multiple putative modes of metformin’s anti-cancer action [213]. Further, Del Barco et al have reviewed the empirical epidemiological evidence for a metformin anti-cancer effect [214] and Chae et al the relevant clinical studies [215].

Across many cancers a large chart review shows decreased mortality in patient’s treated with metformin [216 ] although the effect has not been large. Increased lactate secretion, reduced oxygen consumption, inhibition of mitochondrial inner membrane I complex activity (inner membrane NADH ubiquinone oxidoreductase), and activation of AMPK-signaling have been proposed as mechanisms for metformin’s anticancer effects [217]. To what degree one or the other of these mechanisms is the consequence of one of the other listed mechanisms is unknown. Inhibition of the mitochondrial respiratory chain complex 1 is the leading candidate for a primary mode of anti-cancer action [218]. By inhibiting function of mitochondrial respiratory chain complex 1, mitochondrial ATP production and oxygen consumption are decreased. Compensatory increases in lactate and shift to relative reliance on glycolytic ATP production result in AMPK activation, with mTOR function consequent to that. Decreased proliferation, cell cycle arrest, autophagy, apoptosis and other forms of cell death would then follow from this ensemble.

AMPK is a ubiquitous heterotrimeric protein acting as a kinase with multiple identified targets [219]. Metformin, through inhibition of the mitochondrial respiratory chain complex 1, activates AMPK [220, 221] that in turn suppresses several transcription factors including Snail and Slug [28, 222]. Circulating leukocytes of metformin-treated diabetes patients had hypomethylated E-cadherin promoters and increased E-cadherin levels [28]. Metformin specifically inhibits TGFbeta -induced EMT in non-small cell lung cancer cell lines [223]. Independent studies showed that metformin inhibited TGF-beta-stimulated loss of E-cadherin and gain of vimentin [106, 224] as well the decrease of N-cadherin and prostate cancer cell motility [224].

Decreasing N-cadherin expression can also be an AMPK-independent mode of metformin action in reversing or inhibiting EMT [225]. TGF-beta activated intracellular transcription factors Snail and Twist and cervical cancer EMT are also diminished after metformin [106, 226]. Human endometrial cancer biopsy tissue from patients on metformin had more E-cadherin compared to patients on other anti-diabetes agents [227]. This bodes well for our use of metformin to inhibit EMT during primary GB treatment.

Although tumor bed radiation after GB resection is standard treatment of GB and has proven survival benefit, radiation does participate in enhancing residual GB cell migration as outlined in Section 2.C. above. Metformin inhibits radiation induced EMT features and increased motility in esophageal squamous cell carcinoma cells where metformin also reduced radiation-induced expression of mesenchymal markers vimentin and N-cadherin and reduced transcription factors Slug, Snail, and Twist [228]. In a parallel manner, exposure to docetaxel decreases prostate cancer cells’ mitosis rate, but increases motility, lowers E-cadherin expression, increases MMP-9 and changes morphology typical of EMT. Metformin partially reversed this docetaxel-induced increased motility, lowered E-cadherin, and increased MMP-9 [229].

Metformin also reduced EMT phenotypic changes observed in lung adenocarcinoma cells exposed to TGF-beta, by decreasing Snail2, Twist, and vimentin expression, while increasing E-cadherin [230]. Similarly, metformin also increased E-cadherin, decreased N-cadherin and MMP-9 in a xenografted mouse gastric cancer model [231]. In melanoma cells metformin partially reversed EMT and *in vitro* colony formation triggered by low pH [232]. Metformin increased E-cadherin and phosphorylated AMPK, while decreasing N-cadherin in hepatocellular carcinoma cells [233].

Triple negative breast cancer (TNBC, cells not expressing estrogen receptor, progesterone receptor, or HER2) are particularly aggressive. In these patients, elevated TGF-beta production further worsens the patient’s prognosis. *In vitro*, metformin attenuated the TGF-beta-stimulated TNBC cell growth, invasion and motility [234]. Metformin inhibited EMT in cell lines of thyroid [235], non-small cell lung [236] and prostate cancer [237], and reversed EMT in non-small cell lung [238], breast [239] and prostate cancer [240]. In melanoma, metformin increased E-cadherin and inhibited cell motility, migration and invasion [241].

Experimental renal ischemia-reperfusion results in tubulointerstitial fibrosis accompanied by increased TGF-beta, IL-6, and vimentin with decreased E-cadherin. All these, changes may be partially reversed by metformin [242]. In proximal tubular epithelial cells TGF-beta1 treatment causes a decrease in AMPK phosphorylation and activation together with increased fibronectin and alpha-smooth muscle actin expression and decrease in E-cadherin. Metformin inhibited these TGF-beta induced changes by increasing phosphorylated AMPK [243].

Metformin reversed 17beta-estradiol-induced EMT in endometrial adenocarcinoma cells via an AMPK activating step [222]. Activation of AMPK by metformin inhibited TGF-beta-induced Smad2/3phosphorylation, increase in IL-6 and fibronectin in cancer cells [244], indicative of EMT inhibition. AMPK activation reduces DNA promoter activity resulting downstream to TGF-beta1 and a slight decrease in serum TGF-beta in diabetic patients [245].

In non-small cell lung cancer cells, the inverse relationship between rising IL-6 expression and falling E-cadherin expression was inhibited by metformin [236]. TGF-beta induced EMT in prostate cancer cells, increasing E-cadherin, vimentin and Slug, effects mitigated by metformin [246]. Thus metformin is a promising partner drug for pirfenidone.

As positive data accrues, using metformin as an adjunct to cancer therapies becomes a more and more attractive strategy. For instance, in hepatocellular carcinoma cells metformin alone gave considerable growth inhibition and augmented cytotoxicity of cytotoxic drugs 5-FU, doxorubicin, cisplatin increases oxidative stress synergistically leading to increased apoptosis in treated cells [247].

Metformin was synergistic with sorafinib in increasing intracellular ROS, decreasing efflux pump activity and increasing apoptosis in glioma cells [248]. That metformin augmented temozolomide GIC killing in GB explant culture was shown in 2011 [249] and again independently in 2016 [250]. Metformin’s cytotoxic activity to glioma cells is somewhat selective for the GIC subpopulation [251]. Metformin inhibits glioma cell proliferation at lower concentrations and proliferation plus migration at higher concentrations [252]. Metformin exposure enhanced cytotoxic effects to glioma cells of either temozolomide or radiation and retarded glioma xenograft growth [253]. By progressive temozolomide exposure over 9 months, glioma cells resistant to temozolomide were developed. Exposure to metformin several days before exposure to temozolomide more than reverted these cells’ sensitivity to temozolomide. The authors confirmed metformin retardation of glioma growth in a xenograft model [254].

Leidgens et al found glioma cell exposure to metformin resulted in decreased glioma cell proliferation and increased AMPK activation, as found by others, but also inhibition phosphorylation of STAT3 in [255].

Despite being rather hydrophilic, metformin achieves approximately equal plasma and brain tissue levels. In rats after single dose oral metformin administration, 28 micromol/l plasma and 14 micromol/kg brain tissue were measured [256]. The range of metformin plasma levels typically seen in asymptomatic diabetes patients is unusually wide for a therapeutic drug, reflecting its safety [257]. Of considerable additional potential benefit, metformin reduced glioma-induced brain edema in a rat model [258].

## SAFETY

### Pirfenidone

In clinical practice pirfenidone often has no side effects. Mild nausea in 23%, rash /photosensitivity in 20% (treated minus placebo) of patients were most common side effects when treating idiopathic pulmonary fibrosis [259]. These side effects were generally well tolerated.

### Quetiapine

Quetiapine has a circulating half-life of 7 hours but its dopaminergic (at D2) and serotonergic (at 5-HT2A) receptor antagonistic occupancy is transient. It has significant antidepressant and antipsychotic properties for both of which it is in wide use world wide [260]. Quetiapine is quite well tolerated also in non-psychiatric populations. Some daytime sedation is common after starting quetiapine but this usually wears off after several day’s use. It should only be given once daily at bedtime. This day sedation commonly recurs after each dose increase but again wears off after several day’s use. Importantly for a drug with anti-psychotic properties, quetiapine-related extrapyramidal signs and symptoms do not differ from placebo [260].

### Rifampin

Rifampin has a 4 hr half-life and is relatively safe at doses under 1.2 g/day, under which adverse or side effect risk is small, although in rare cases serious [261]. Drug-induced hepatitis is recognized as a rare possibility. Since rifampin is a strong inducer of P450 3A4 [262] and use with a strong 3A4 inhibitor, itraconazole, is envisioned, the net effect will be unclear so caution would be required when using other drugs that may be influenced by this catabolizing system. Rifampin also strongly increases level and activity of CYP2A6, CYP2C8, 2C9, and 2B6 [262].

### Naproxen

Naproxen is so well tolerated that it is widely available around the world over-the-counter (i.e. available without a prescription), the most common side effect being gastrointestinal irritation and microbleeds. Stomach or duodenal ulceration occurs rarely. Prior H.pylori eradication and administration with a proton pump inhibitor will reduce that risk even further. Naproxen is a balanced COX1/COX2 inhibitor, has a 14 hr half-life, is metabolized by multiple hepatic P450 enzymes, prominent among which are 1A2, 2C8 and 2C9 and has been marketed since the 1970’s. Naproxen hasn’t the cardiovascular risks of other COX inhibitors and in fact does share with aspirin some of that drug’s cardioprotective antiplatelet function [263].

### Itraconazole

Itraconazole is a lipophilic, 24 hr half-life, broad spectrum anti-fungal drug [264]. Serum levels < 17.1 mg/L are associated with fewer side effects than are higher levels. Out of 9065 patients treated with itraconazole, 17% had mild GI upset, liver function elevation in 5%, rash in 3%, 2% hypokalemia, 1% headache, but only 1 case of hepatitis, with multiple rare cases of various other side effects [264]. If a proton pump inhibitor is concomitantly used, itraconazole must be given with orange or lemon juice to achieve good absorption.

### Metformin

Most people starting metformin experience no side effect. Metformin is one of the safer anti-diabetic drugs as it does not induce hypoglycemia. Mild and transient GI upset occurs in 5% of non-insulin-dependent diabetes patients starting metformin. Rare lactic acidosis has been reported [265, 266]. Most of ingested metformin appears unchanged in urine and feces [265, 266].

## CONCLUSIONS

All three arms of current GB treatment - surgery, cytotoxic chemotherapy with temozolomide, and radiation - all tend to engage EMT programs. Post-EMT GB cells are more aggressive, more treatment resistant, and are in a high migration, apoptosis resistant state. The EIS Regimen was developed with the aim of limiting this inherent negative consequence of current GB treatment. The EIS Regimen uses only already-marketed drugs, repurposed for their ancillary attributes in inhibiting one or another of the EMT triggers, as described above in Section 3.

As a preliminary step the efficacy of the EIS regimen towards GB might best be investigated in preclinical survival studies on immunodeficient mice bearing GIC-driven orthotopic tumors. This is presently the most reliable and informative animal model we have - albeit an imperfect one- for exploring the efficacy of novel treatments against GB, prior to clinical Phase I/II studies.

In the meantime, given the lethality of GB, the ubiquity of recurrence after primary treatment, the paucity of treatments we have to offer after recurrence, it could be reasonable to add the EIS Regimen to initial current standard in a small pilot study to test the hypothesis of this paper, that the EIS Regimen with inhibit treatment-induced EMT and thereby delay recurrence. Blocking EMT with the EIS Regimen was designed to be best applied peri-operatively, even before first biopsy, and used continuously during radiation and primary treatment with temozolomide for at least one year.

This paper proposes the simple idea that, by combining individual interventions that might be weak individually, we may get an additive therapeutic effect, particularly when the drug effects are coordinated as we have attempted to do. The expected safety profile of the ensemble of EIS Regimen drugs is based on the well-known safety profile of the individual drugs.

We are fully aware that some other drivers of EMT that have been identified are not addressed by EIS Regimen. Functional redundancy may be a major problem when trying to pharmacologically block malignancy-promoting pathways and processes like EMT, given its expected safety and the usual outcome of GB as it stands today, the EIS Regimen is a rational option that we might explore with profit today using today’s tools.

### Highlights

- EMT is an element of malignant process where tumor cells become loose, more motile but less mitotic and less sensitive to cytotoxic drugs and radiation
- EMT is a central initial process in establishment of metastases or tumor spreading in the organ of primary occurrence such as brain.
- Standard cancer treatment modalities- cytotoxic chemotherapy, radiation and surgical resection, all tend to provoke or increase EMT.
- Six older drugs- itraconazole, metformin, naproxen, pirfenidone, quetiapine, and rifampin have good preclinical evidence that they can be repurposed to interfere with EMT process and therefore be of adjunctive benefit when treating cancer generally, glioblastoma specifically, to run alongside of standard current treatment.

## Abbreviations

AMPK: AMP kinase
CSF: cerebrospinal fluid
COX: cyclooxygenase
EGFR: epidermal growth factor receptor
EMT: epithelial to mesenchymal transition
EIS: epithelial to mesenchymal transition inhibiting sextet
GB: glioblastoma
GIC: glioma initiating cells
Hh: Hedgehog signaling
HIF: Hypoxia Inducible Factors
MMP: matrix metalloproteinase
MET: mesenchymal to epithelial transition
miRNA: microRNA
PDGF: platelet derived growth factor
RANKL: receptor activator of NFkB ligand
TGF-beta: transforming growth factor-beta
TNBC: triple negative breast cancer
VEGFR: vascular endothelial growth factor receptor
